# Supplementary material for: Multivariate genomic scan implicates novel loci and haem metabolism in human ageing
Source: Nat Commun. 2020 Jul 16;11:3570. doi: 10.1038/s41467-020-17312-3 (PMC7366647; doi:10.1038/s41467-020-17312-3)
Supplement: Supplementary file 1 — Supplementary Information [file 41467_2020_17312_MOESM1_ESM.docx]

Multivariate genomic scan implicates novel loci and haem metabolism in human ageing

Timmers et al.


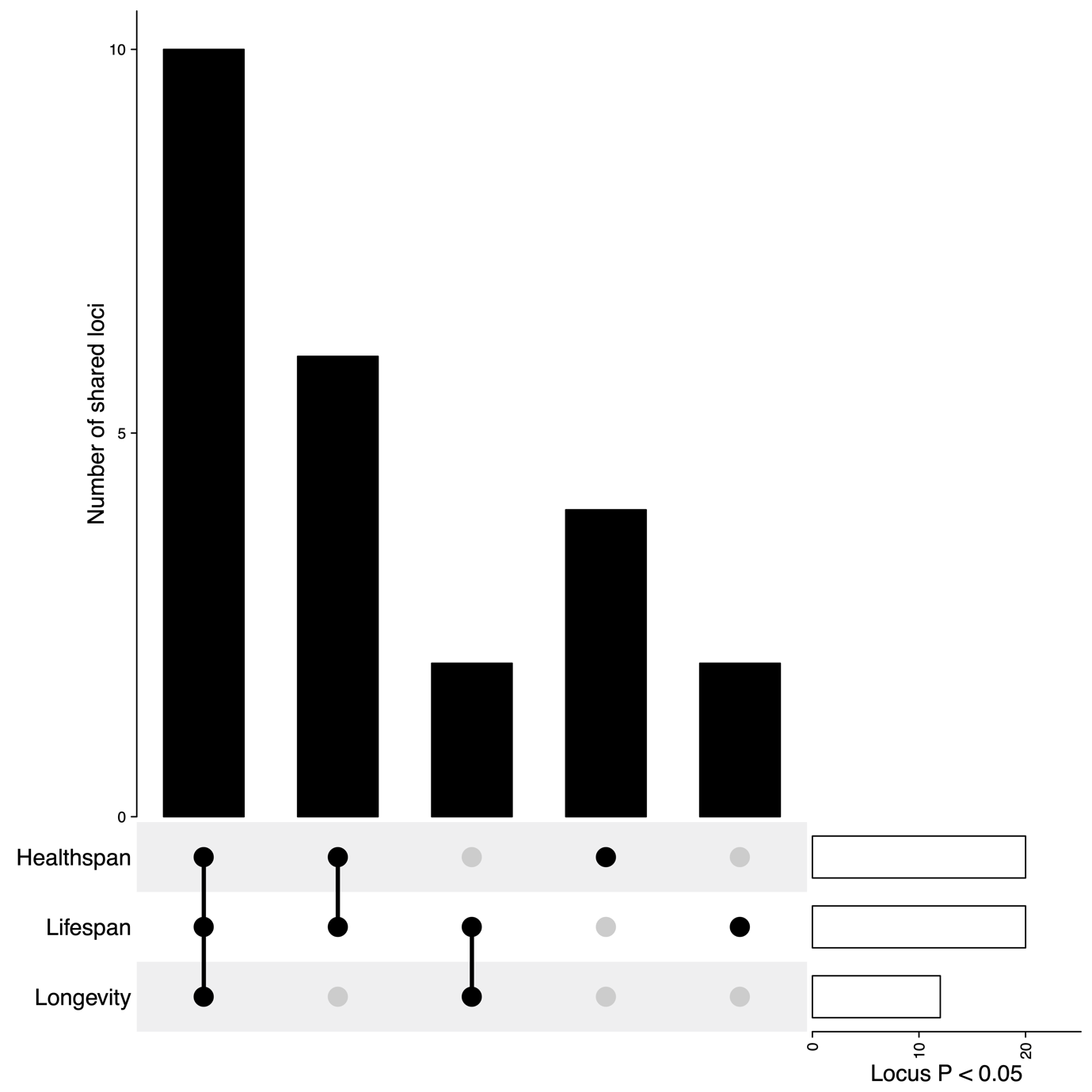


**Supplementary Figure 1: Association of lead multivariate SNPs with ageing traits.** Lead genome-wide significant SNPs from the multivariate GWAS are shown here grouped by their nominal association (two-sided P < 0.05) with the healthspan, lifespan, and longevity GWAS. Bars on the right represent the number of lead SNPs reaching nominal significance in the individual healthspan, lifespan, and longevity GWAS, while bars on top represent the number of SNPs reaching nominal significance across studies.


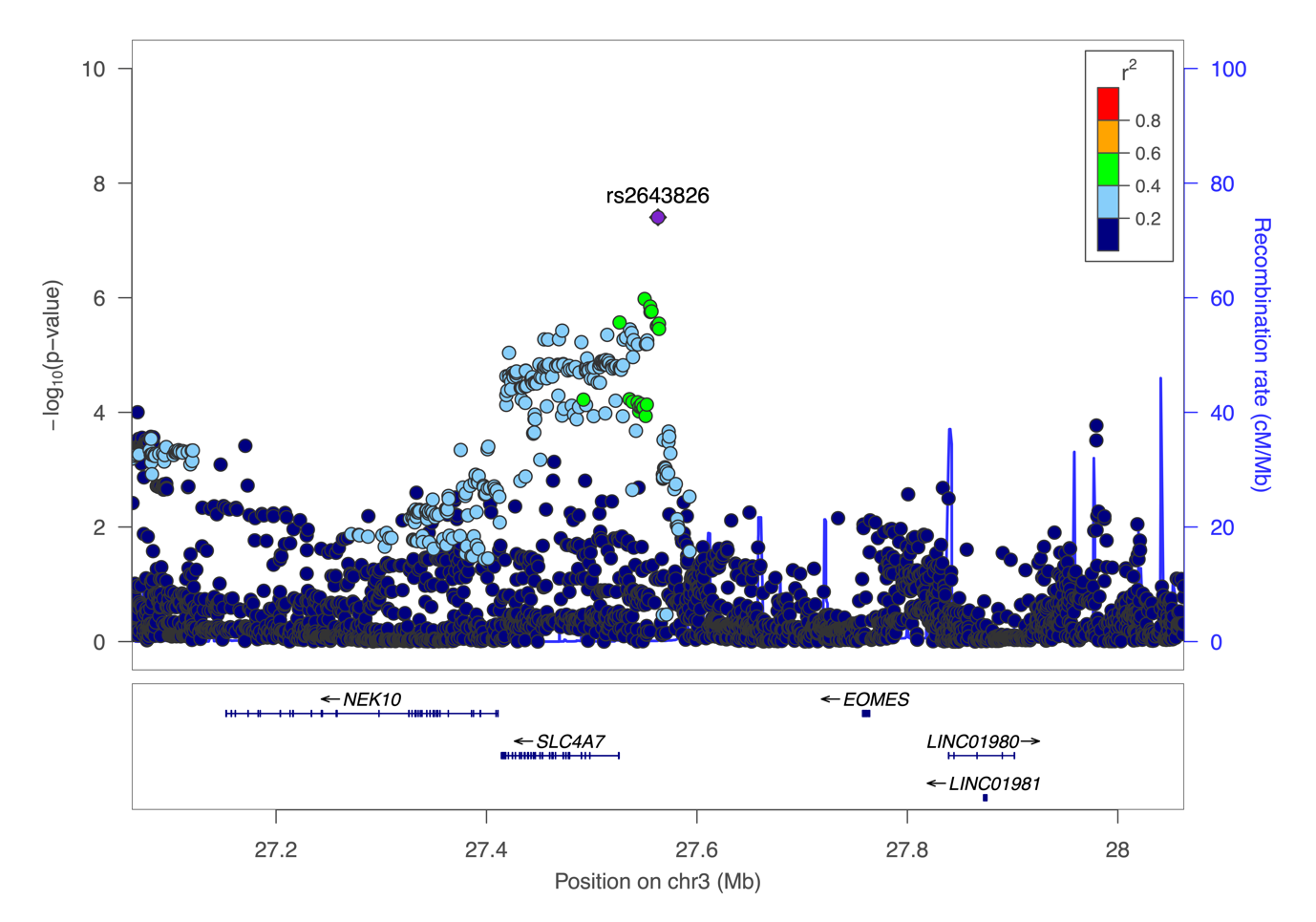


**Supplementary Figure 2: LocusZoom plots of rs2643826 near *SLC4A7.*** The x-axis shows the base-pair position of SNPs on chromosome 3 (GRCh37). The y-axis shows the nominal strength of the MANOVA association (two-sided P value). SNPs are coloured by their degree of linkage disequilibrium with the lead SNP, based on 1000 Genomes European ancestry individuals.


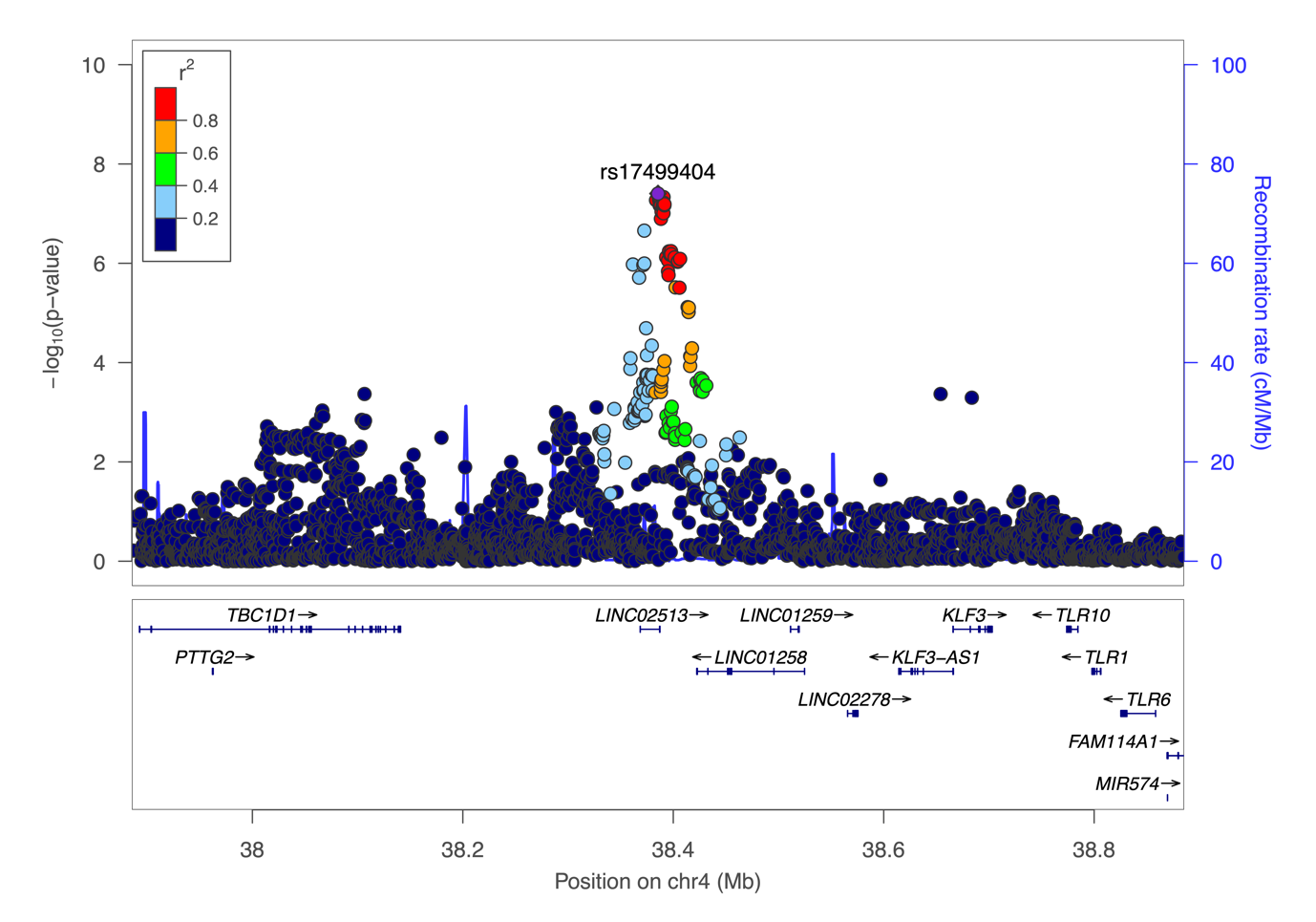


**Supplementary Figure 3: LocusZoom plots of rs17499404 near *LINC02513.*** The x-axis shows the base-pair position of SNPs on chromosome 4 (GRCh37). The y-axis shows the nominal strength of the MANOVA association (two-sided P value). SNPs are coloured by their degree of linkage disequilibrium with the lead SNP, based on 1000 Genomes European ancestry individuals.

**
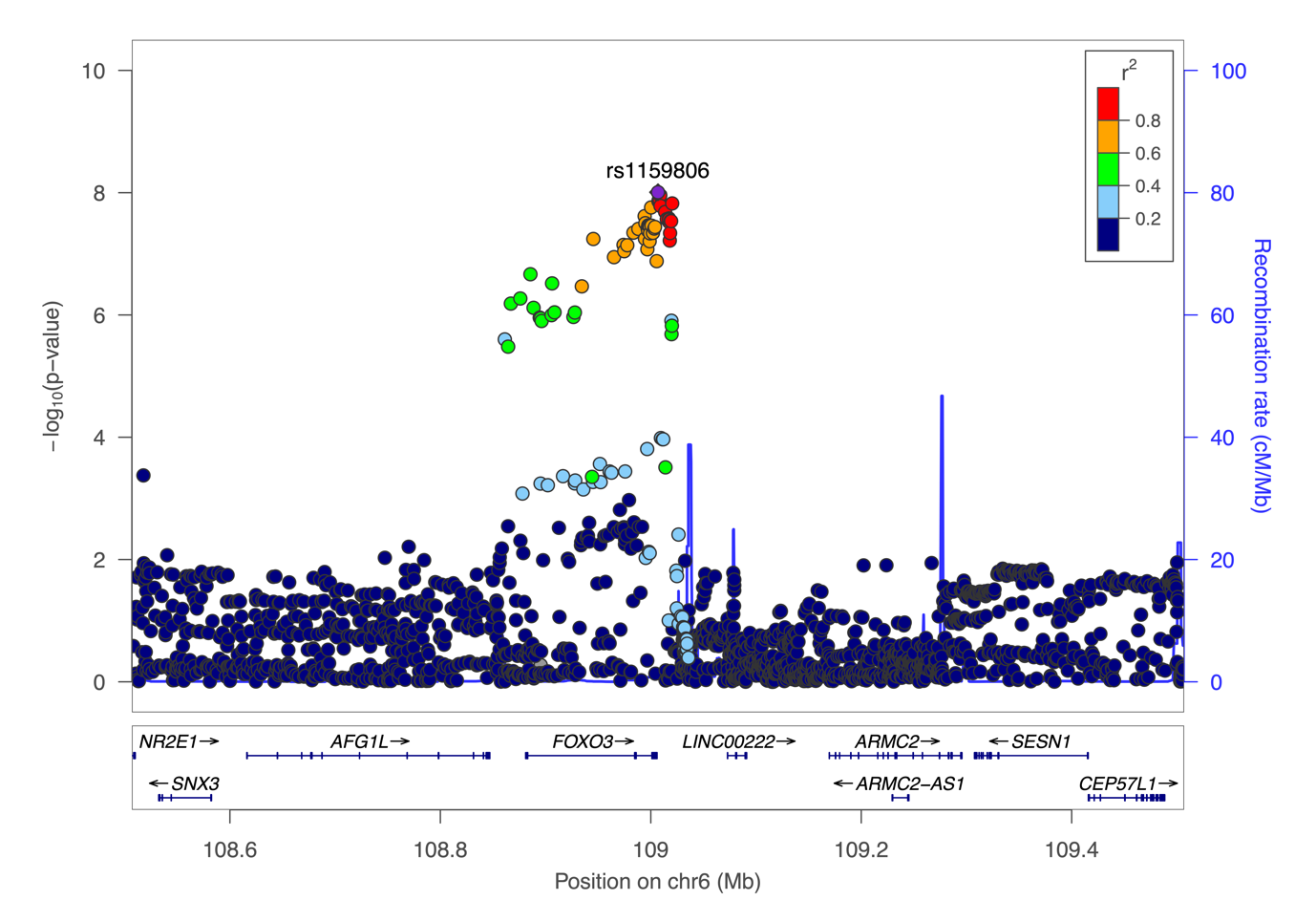
**

**Supplementary Figure 4: LocusZoom plots of rs1159806 near *FOXO3.*** The x-axis shows the base-pair position of SNPs on chromosome 6 (GRCh37). The y-axis shows the nominal strength of the MANOVA association (two-sided P value). SNPs are coloured by their degree of linkage disequilibrium with the lead SNP, based on 1000 Genomes European ancestry individuals.


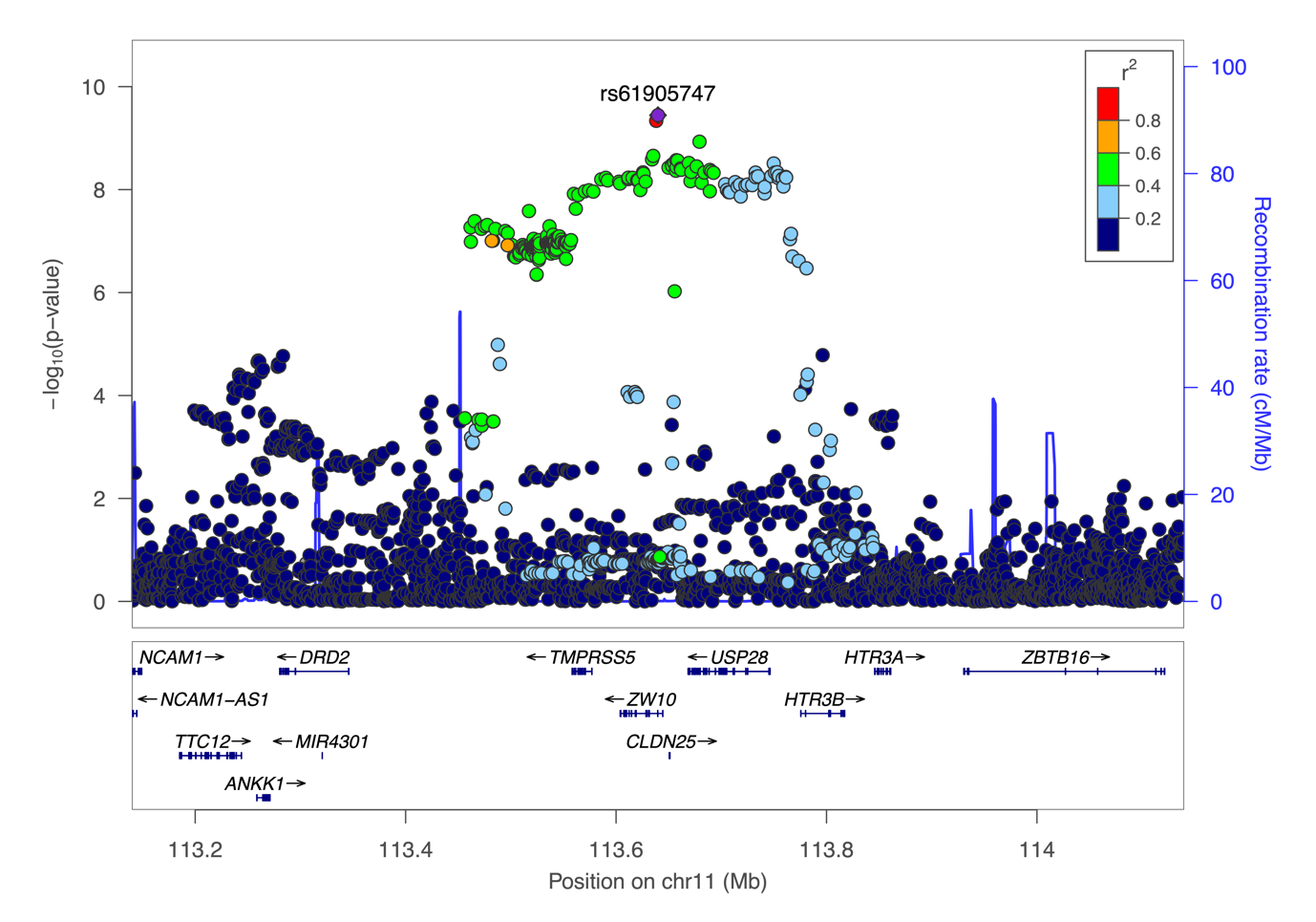


**Supplementary Figure 5: LocusZoom plots of rs61905747 near *ZW10.*** The x-axis shows the base-pair position of SNPs on chromosome 11 (GRCh37). The y-axis shows the nominal strength of the MANOVA association (two-sided P value). SNPs are coloured by their degree of linkage disequilibrium with the lead SNP, based on 1000 Genomes European ancestry individuals.


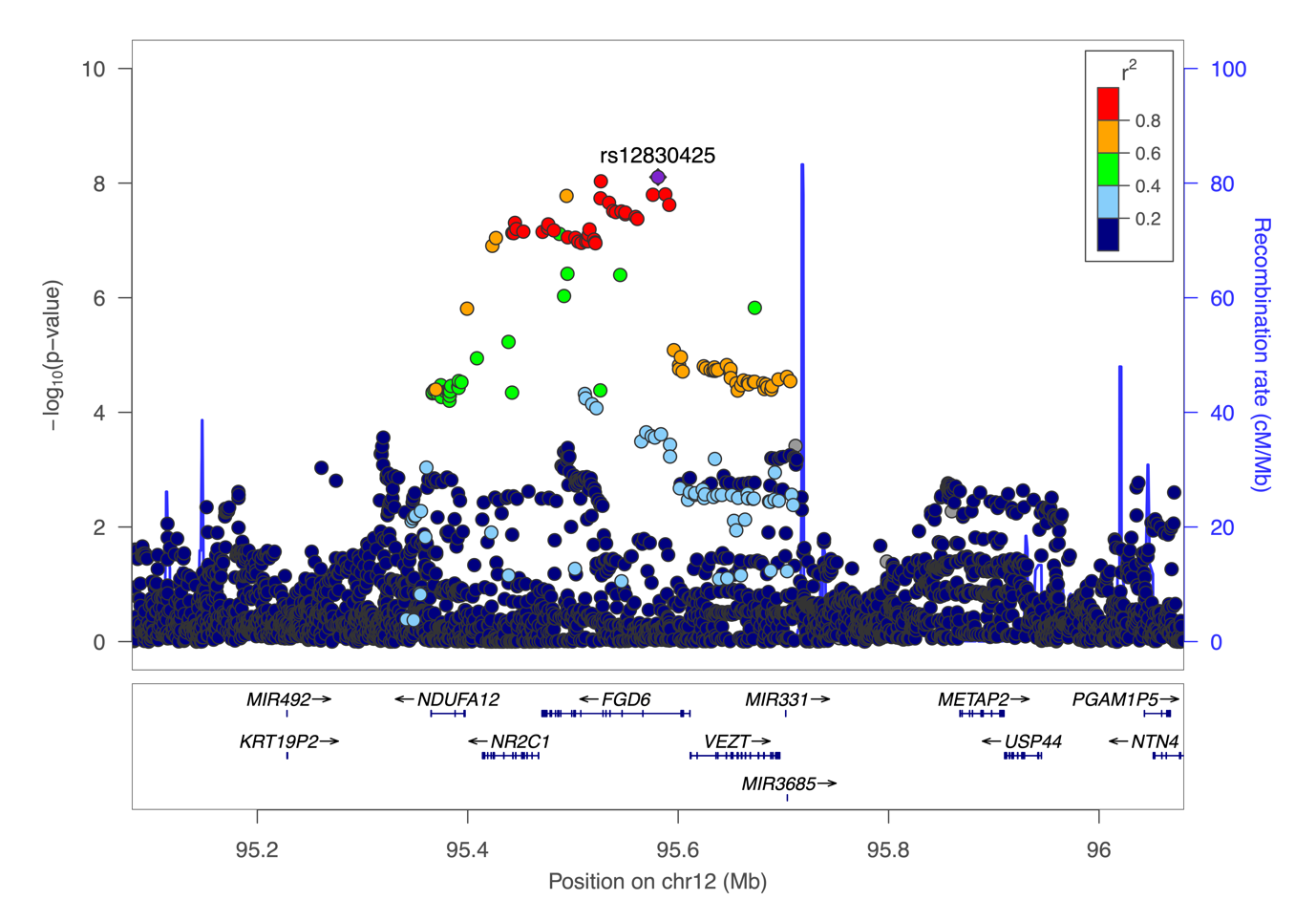


**Supplementary Figure 6: LocusZoom plots of rs12830425 near *FGD6.*** The x-axis shows the base-pair position of SNPs on chromosome 12 (GRCh37). The y-axis shows the nominal strength of the MANOVA association (two-sided P value). SNPs are coloured by their degree of linkage disequilibrium with the lead SNP, based on 1000 Genomes European ancestry individuals.


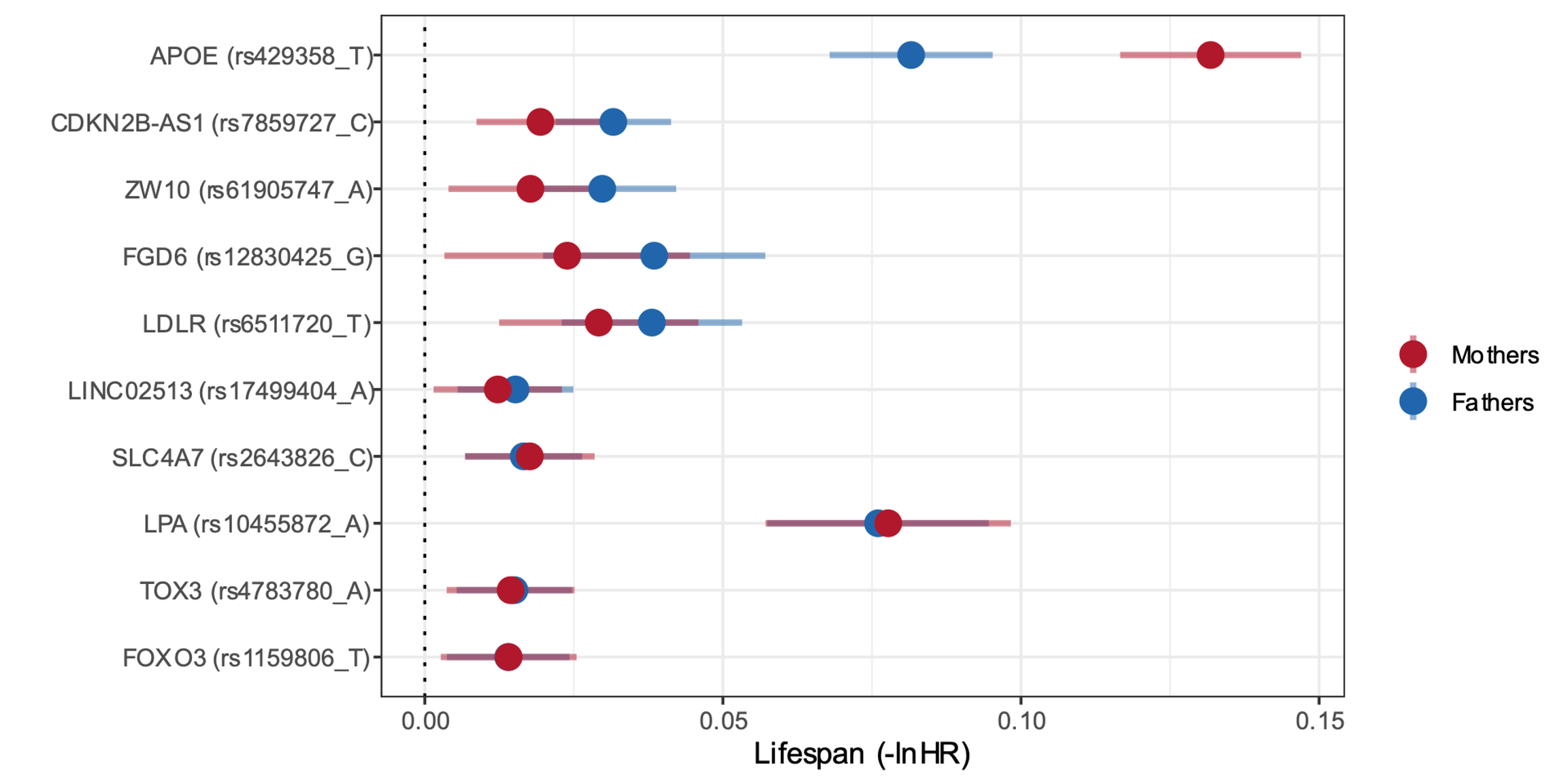


**Supplementary Figure 7: Apart from *APOE*, loci of interest show limited evidence of sex specificity.** Shown here are the effects from Timmers et al. (2019) for carrying one copy of the lead SNP for each locus of interest on mother and father survival, in red and blue, respectively. These were calculated from the lifespans of 512,047 mothers and 500,193 fathers of individuals from UK Biobank. The units are negative log hazard ratios, with higher values indicating longer life. Annotated for each locus are the nearest gene, index SNP and lifespan-increasing allele. Lines represent 95% confidence intervals of the effect estimate.

**
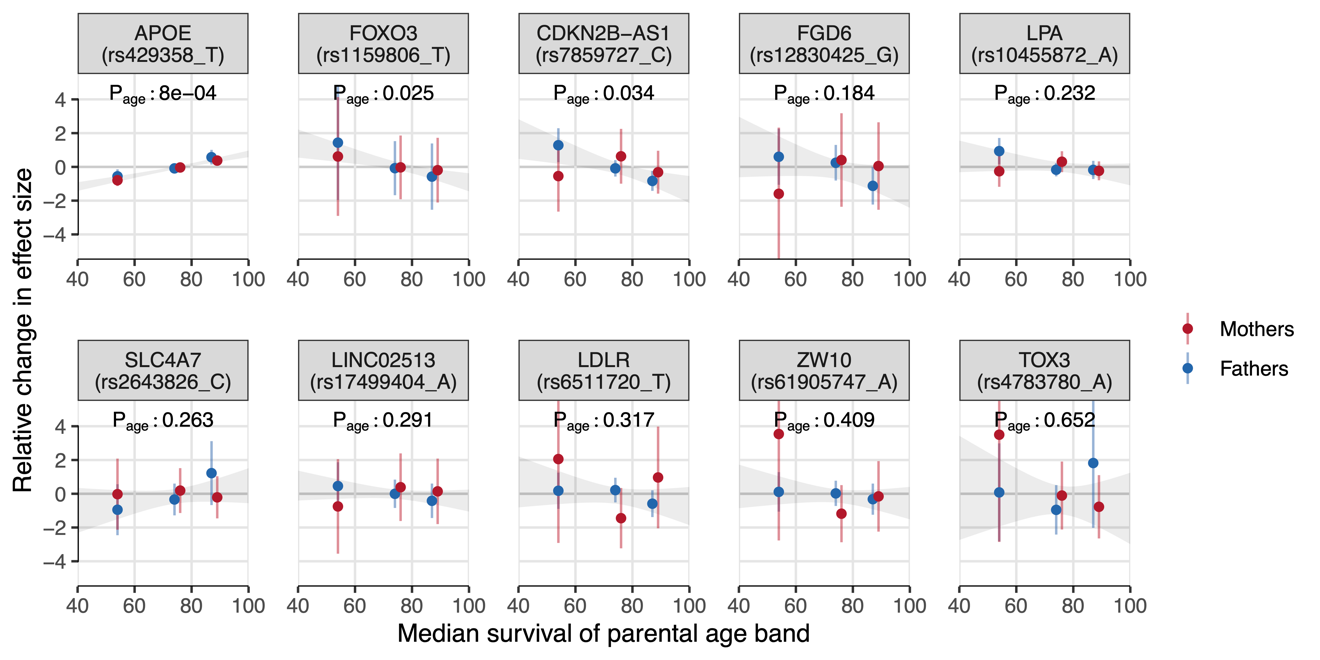
**

**Supplementary Figure 8: Apart from *APOE*, most loci of interest tend to decrease in effect with age.** Each panel contains the age-stratified estimates of the lead SNP in each locus of interest on parental survival, annotated with the nearest gene, lead SNP ID and its protective allele. The y-axis shows the effect sizes of age-stratified analyses (parents survival 40-60, 60-80, and 80+) relative to the unstratified analysis, for mothers and fathers separately. Lines represent 95% confidence intervals. The x-axis shows the median survival of each parental age band, calculated from Kaplan-Meier curves. See Supplementary Table 4 for the number of individuals and deaths of each age band and their median survival. Grey ribbons indicate the regression of father and mother estimates against median survival, weighted by the inverse variance of each estimate. The annotated P_age_ value is the nominal two-sided P value of the coefficient of median survival in this regression (only *APOE* remains significant after multiple testing correction).

**Supplementary Figure 9: Loci of interest have previously been linked to cardiovascular traits**. This heatmap shows the number of genome-wide significant associations reported in the GWAS catalog and PhenoScanner for lead SNPs and close proxies (r^2^_EUR_ > 0.6) of each locus of interest. Loci annotated with green bars have not been reported at genome-wide significance in the healthspan, parental lifespan, and longevity GWAS, while those annotated with grey bars reach this threshold in at least one study.

**Supplementary Figure 10: Genes of interest are enriched (P_bonferroni_ < 0.05) for biological processes related to apoptosis and chemical homeostasis.** Genes colocalizing with loci of interest in cis or trans are listed on the x-axis; GO biological processes gene sets from the Molecular Signatures Database, grouped into 8 broad categories using k-means clustering, are listed on the y-axis. See Supplementary Data 7 for the full list of 32 biological process pathways with hypergeometric P < 0.05/383 contained in these categories, where 383 is the number of gene sets passing the inclusion criteria. Squares represent the presence of a gene within one or more gene sets contained in the broad category. Squares are coloured based on their colocalisation with loci of interest.


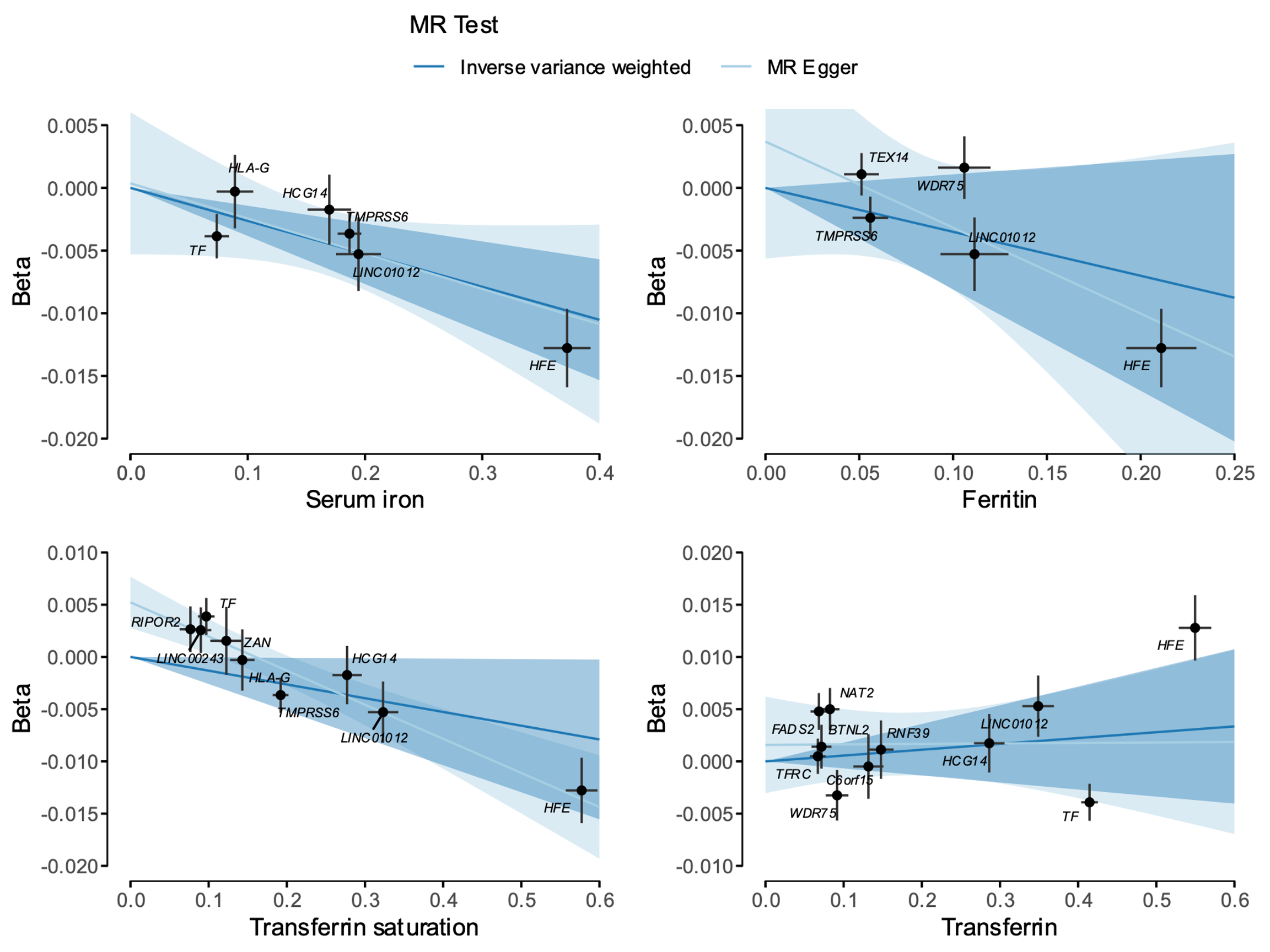


**Supplementary Figure 11: Serum iron and transferrin saturation may influence healthy life.** Each panel contains the inverse variance-weighted MR and MR Egger regressions of SNP effects from iron-related GWAS against the effects of the same SNPs within our multivariate GWAS. See Supplementary Tables 2 and 3 for numeric details. Dots represent lead genome-wide significant iron-related SNPs, annotated with the nearest gene and error bars representing the standard error of the effect estimate. Lines represent the regression slope and ribbons the 95% confidence intervals of the regression. Standardised effects of the SNPs on the iron-related traits are listed on the x-axis, which were estimated by Benyamin et al. (2014) based on 48,972 individuals. Derived effects of the SNPs on the multivariate GWAS are listed on the y-axis (Beta), assuming an effective sample size of 709,709 individuals. Note the scales vary between panels.

|  |  |  | Number of SNPs in common | | |
| --- | --- | --- | --- | --- | --- |
| Trait | PMID | h² (SE) | Healthspan | Lifespan | Longevity |
| Age at menarche | 28436984 | 0.160 (0.008) | 1,180,531 | 1,020,213 | 1,148,951 |
| Age at menopause | 26414677 | 0.136 (0.016) | 1,051,150 | 932,115 | 1,031,763 |
| Allergic disease | 29083406 | 0.072 (0.006) | 1,167,425 | 1,016,121 | 1,139,769 |
| Alzheimer's disease | 29777097 | 0.069 (0.032) | 1,175,362 | 1,017,945 | 1,148,143 |
| Male pattern baldness | 29146897 | 0.678 (0.106) | 1,177,695 | 1,017,854 | 1,146,251 |
| Body mass index | 30239722 | 0.205 (0.007) | 1,172,575 | 1,018,624 | 1,141,876 |
| Breast cancer | 30349118 | 0.010 (0.002) | 1,169,354 | 1,016,398 | 1,141,305 |
| Colorectal cancer | 30104761 | 0.246 (0.060) | 1,181,238 | 1,020,218 | 1,148,977 |
| Digestive cancers | 30349118 | 0.004 (0.001) | 1,169,354 | 1,016,398 | 1,141,305 |
| Prostate/Testicular cancer | 30349118 | 0.011 (0.001) | 1,169,354 | 1,016,398 | 1,141,305 |
| Lymphoid cancer | 30349118 | 0.001 (0.001) | 1,169,354 | 1,016,398 | 1,141,305 |
| Melanoma | 30349118 | 0.022 (0.004) | 1,169,354 | 1,016,398 | 1,141,305 |
| Lung cancers | 30349118 | 0.002 (0.001) | 1,169,354 | 1,016,398 | 1,141,305 |
| Urinary cancers | 30349118 | 0.001 (0.001) | 1,169,354 | 1,016,398 | 1,141,305 |
| COPD | 30349118 | 0.035 (0.003) | 1,169,354 | 1,016,398 | 1,141,305 |
| Coronary artery disease | 21378990 | 0.188 (0.024) | 940,303 | 866,405 | 923,773 |
| Depression | 30718901 | 0.135 (0.005) | 1,159,475 | 1,012,345 | 1,131,616 |
| Alcohol intake | 31358974 | 0.067 (0.003) | 1,173,945 | 1,019,665 | 1,146,709 |
| Hernia | 30349118 | 0.023 (0.002) | 1,169,354 | 1,016,398 | 1,141,305 |
| Inflammatory bowel disease | 26192919 | 0.693 (0.077) | 1,077,162 | 1,000,713 | 1,055,914 |
| Pubertal growth | 23449627 | 0.430 (0.048) | 1,008,508 | 913,619 | 990,765 |
| Rheumatoid arthritis | 21378990 | 1.105 (0.197) | 1,171,270 | 1,019,352 | 1,144,146 |
| Risk taking | 30271922 | 0.126 (0.006) | 1,173,834 | 1,019,629 | 1,146,612 |
| Ever smoked | UKBB_Neale | 0.075 (0.003) | 1,176,233 | 1,017,808 | 1,144,805 |
| Stroke | 29531354 | 0.013 (0.001) | 1,177,239 | 1,020,193 | 1,148,764 |
| Type 2 diabetes | 30054458 | 0.053 (0.003) | 1,006,740 | 935,569 | 991,467 |
| Years of schooling | 21378990 | 0.115 (0.003) | 1,172,617 | 1,015,857 | 1,141,715 |

**Supplementary Table 1: Technical details of 27 European-ancestry GWAS of traits related to development, behaviour, and disease.** PMID—PubMed identification number, where UKBB_Neale refers to the analysis of UK Biobank traits by Neale Lab (Abbott et al., 2018); h^2^—SNP-based heritability of the trait, as calculated by LD-score regression; SE—Standard error of the heritability. Final three columns are the number of high-quality (INFO > 0.9; MAF ≥ 0.05; MHC excluded) HapMap3 SNPs in common between the trait GWAS and the healthspan, parental lifespan, and longevity GWAS, respectively.

|  |  |  |  | MR IVW | | MR Egger | |
| --- | --- | --- | --- | --- | --- | --- | --- |
| Exposure | N SNP | Beta | SE | P | FDR | P | FDR |
| Serum Iron | 6 | -0.03 | 0.005 | 4E-07 | 3E-06 | 0.067 | 0.090 |
| Transferrin saturation | 8 | -0.02 | 0.004 | 6.E-04 | 0.002 | 0.003 | 0.009 |
| Ferritin | 5 | -0.04 | 0.017 | 0.034 | 0.068 | 0.112 | 0.128 |
| Transferrin | 9 | 0.01 | 0.007 | 0.050 | 0.081 | 0.683 | 0.683 |

**Supplementary Table 2: Details of univariate Mendelian Randomisation analyses.** Exposure—The GWAS statistics used as exposure in the MR analysis. N SNP—total number of SNPs used as instrumental variables. Beta—Regression slope of SNP effects from the exposure GWAS against the SNP effects in our multivariate GWAS. SE—Standard Error. P—nominal two-sided P value of the regression. FDR—Benjamini-Hochberg-adjusted P value.

| Exposure | Excluding SNP | SNP Label | Beta | SE | P |
| --- | --- | --- | --- | --- | --- |
| Serum Iron | rs13217599 | LINC01012 | -0.03 | 0.006 | 3E-06 |
| Serum Iron | rs1525892 | TF | -0.03 | 0.005 | 3E-06 |
| Serum Iron | rs1800562 | HFE | -0.02 | 0.007 | 1E-03 |
| Serum Iron | rs3115630 | HLA-G | -0.03 | 0.005 | 3E-07 |
| Serum Iron | rs3118362 | HCG14 | -0.03 | 0.005 | 3E-07 |
| Serum Iron | rs855791 | TMPRSS6 | -0.03 | 0.006 | 3E-06 |
| Serum Iron | None | - | -0.03 | 0.005 | 4E-07 |
| Ferritin | rs12693541 | WDR75 | -0.05 | 0.015 | 1E-03 |
| Ferritin | rs13217599 | LINC01012 | -0.03 | 0.020 | 1E-01 |
| Ferritin | rs1800562 | HFE | -0.01 | 0.018 | 5E-01 |
| Ferritin | rs2413450 | TMPRSS6 | -0.03 | 0.020 | 9E-02 |
| Ferritin | rs368243 | TEX14 | -0.04 | 0.017 | 1E-02 |
| Ferritin | None | - | -0.04 | 0.017 | 3E-02 |
| Transferrin saturation | rs13217599 | LINC01012 | -0.02 | 0.005 | 4E-03 |
| Transferrin saturation | rs1800562 | HFE | -0.01 | 0.005 | 8E-02 |
| Transferrin saturation | rs221834 | ZAN | -0.02 | 0.005 | 5E-04 |
| Transferrin saturation | rs3115630 | HLA-G | -0.02 | 0.005 | 1E-03 |
| Transferrin saturation | rs3118362 | HCG14 | -0.02 | 0.005 | 7E-04 |
| Transferrin saturation | rs3130784 | LINC00243 | -0.02 | 0.004 | 5E-05 |
| Transferrin saturation | rs7759489 | RIPOR2 | -0.02 | 0.004 | 7E-05 |
| Transferrin saturation | rs855791 | TMPRSS6 | -0.01 | 0.005 | 5E-03 |
| Transferrin saturation | None | - | -0.02 | 0.004 | 6E-04 |
| Transferrin | rs13217599 | LINC01012 | 0.01 | 0.009 | 2E-01 |
| Transferrin | rs1495741 | NAT2 | 0.01 | 0.006 | 8E-02 |
| Transferrin | rs174577 | FADS2 | 0.01 | 0.006 | 6E-02 |
| Transferrin | rs2517438 | C6orf15 | 0.01 | 0.007 | 5E-02 |
| Transferrin | rs3118362 | HCG14 | 0.02 | 0.008 | 5E-02 |
| Transferrin | rs744653 | WDR75 | 0.02 | 0.006 | 2E-02 |
| Transferrin | rs9261387 | RNF39 | 0.01 | 0.007 | 7E-02 |
| Transferrin | rs9268633 | BTNL2 | 0.01 | 0.007 | 8E-02 |
| Transferrin | rs9990333 | TFRC | 0.01 | 0.007 | 7E-02 |
| Transferrin | None | - | 0.01 | 0.007 | 5E-02 |

**Supplementary Table 3: Univariate Mendelian Randomisation leave-one-out sensitivity analysis.** Exposure—The GWAS statistics used as exposure in the MR analysis. Excluding SNP—Inverse variance weighted MR analysis without the specified SNP. SNP Label—Nearest gene to the SNP. Beta—Regression slope of SNP effects from the exposure GWAS against the SNP effects in our multivariate GWAS. SE—Standard Error. P—Nominal two-sided P value of the regression.

|  |  |  |  | Age | |  |
| --- | --- | --- | --- | --- | --- | --- |
| Parents | Group | N | Dead | Mean | SD | Survival |
| Fathers | ALL | 312,100 | 246,700 | 73.06 | 11.15 | 76 |
| Fathers | 40-60 | 312,100 | 43,290 | 59.06 | 3.085 | 54 |
| Fathers | 60-80 | 272,900 | 140,300 | 74.21 | 6.058 | 74 |
| Fathers | 80+ | 96,340 | 65,010 | 85.09 | 4.04 | 87 |
| Mothers | ALL | 322,700 | 192,500 | 76.53 | 10.79 | 83 |
| Mothers | 40-60 | 322,700 | 25,210 | 59.45 | 2.461 | 54 |
| Mothers | 60-80 | 300,200 | 96,170 | 75.57 | 5.652 | 76 |
| Mothers | 80+ | 141,200 | 80,770 | 85.95 | 4.438 | 89 |

**Supplementary Table 4: Sample descriptives of the age-stratified UK Biobank cohort.** We split parental lifespan statistics reported by UK Biobank individuals into age bands, excluding any parent who died before the start of the band and treating any parent still alive at the end of the band as censored. N—Number of parents included in the age band. Dead—Number of parents who died within the span of the age band. Age—Parent ages within the span of the age band. SD—Standard deviation. Survival—Median age at death of the parents within the span of the age band.
