# Supplementary material for: Multivariate genomic scan implicates novel loci and haem metabolism in human ageing
Source: Nat Commun. 2020 Jul 16;11:3570. doi: 10.1038/s41467-020-17312-3 (PMC7366647; doi:10.1038/s41467-020-17312-3)
Supplement: Supplementary file 3 — Descriptions of Additional Supplementary Files [file 41467_2020_17312_MOESM3_ESM.pdf]

Descriptions of Additional Supplementary Files

Supplementary Data 1

Description: Genetic correlations of 27 European-ancestry GWAS with healthspan, parental lifespan, and longevity GWAS. PubMed identification number, where UKBB_Neale refers to the analysis of UK Biobank traits by Neale Lab (Abbott et al., 2018). Correlation—Genetic correlation calculated by LD-score regression; SE— Standard Error; P—Nominal P value for the correlation. P Het—Nominal P value for Cochran's Q heterogeneity between ageing GWAS correlation estimates.

Supplementary Data 2

Description: Twenty-four loci identified at genome-wide significance in the multivariate analysis. Nearest gene—Gene closest to the index SNP; rsID—The index SNP with the lowest P value in the multivariate analysis. Chr—Chromosome; Position—Base-pair position on chromosome (GRCh37); A1—the effect allele; Freq1— Frequency of the A1 allele. Beta1—Effect size of the A1 allele, for healthspan and lifespan this is the negative log of the hazard ratio, for longevity this is the log odds of reaching an exceptional old age (90th percentile). SE—Standard error of the effect estimate. P—Nominal two-sided P value of the SNP-trait association. MANOVA P—Nominal two-sided P value against the null hypothesis of association with neither healthspan, lifespan, nor longevity.

Supplementary Data 3

Description: Known associations of lead SNPs of multivariate loci. Genome-wide significant associations reported in the GWAS catalog and PhenoScanner are listed for lead SNPs and close proxies (r2 EUR > 0.6) of each locus of interest. Similar associations have been grouped, keeping the most significant association and the shortest trait name (Trait). Locus—Nearest gene to lead SNP in the locus. Lead rsID—Reference SNP cluster ID for the most significant variant in the locus. A1—The effect allele, associated with an increase in healthspan, lifespan, and/or longevity. A0—The reference allele. Proxy rsID—Reference SNP cluster ID of the proxy SNP. P.A1—The effect allele of the proxy, associated with an increase in healthspan, lifespan, and/or longevity. P.A0—The reference allele of the proxy. R2—Degree of linkage disequilibrium (range 0.60–1.00). Freq1—Frequency of the A1 (proxy) allele in the study, as reported by the GWAS catalog or PhenoScanner. Beta1—The reported effect on the trait of per copy of A1 allele. SE— Standard error of the effect. P—Nominal two-sided P value of the association. PMID— PubMed identification number, where UKBB_Neale refers to the analysis of UK Biobank traits by Neale Lab (Abbott et al., 2018). Category—Disease or risk factor category of the trait, or Miscellaneous, if there is no clear association with a single category

Supplementary Data 4

Description: Supplementary Data 4: Strongest SNPs for each dataset within 500 kb to the lead multivariate SNP. Locus—Nearest gene to the lead SNP in the multivariate analysis. rsID— SNP with the strongest association within the locus. Chr—Chromosome. Position—Base-pair position on chromosome (GRCh37). P—Nominal two-sided P-value for the association of the marker with the trait

Supplementary Data 5

Description: Colocalisation of eQTLs and MANOVA GWAS signals using SMR-HEIDI. Locus—Nearest gene to lead variant in the multivariate analysis. Chr—Chromosome. Position—Base-pair position of the lead variant (GRCh37). Gene— Gene for which the expression in blood colocalizes with the GWAS signal. P_SMR— Nominal two-sided P value for the colocalisation signal. FDR_SMR—BenjaminiHochberg-corrected P value, accounting for multiple testing separately for each eQTL dataset. P_HEIDI—Nominal P value for the heterogeneity of dependent instruments. N_HEIDI—Number of SNPs used for the heterogeneity test. Direction—Direction of the SMR effect estimate, where + indicates higher gene expression is linked to an increase in multivariate traits, and – indicates lower gene expression is linked to an increase in multivariate traits. Tissue—Tissue in which eQTLs were measured. Dataset—eQTL dataset, either Westra (Westra et al., 2013), CAGE (Lloyd-Jones et al., 2017), Vosa (Võsa et al., 2018), or GTEx v7 (GTEx Consortium, 2017).

Supplementary Data 6

Description: Age-related expression of Cis- and Trans-Genes identified by SMR-HEIDI. Locus—Nearest gene to lead variant in the multivariate analysis. Gene— Gene for which the expression in blood colocalizes with the GWAS signal. Cis/Trans— Indication if genes are in close physical proximity (500 kb, Trans) from the lead variant of the locus which colocalise with the multivariate signal. SMR—Direction of the SMR effect estimate, where + indicates higher gene expression is linked to an increase in multivariate traits, and – indicates lower gene expression is linked to an increase in multivariate traits. Direction—Direction of the association with age from Peters et al. (2015), where + indicates higher gene expression with increasing age, and – indicates lower gene expression with increasing age. P—Twosided nominal P value for the association with age as reported in Peters et al. (2015).

Supplementary Data 7

Description: Enrichment of hallmark and biological process gene sets. N—number of genes of interest in the gene set. N set—total number of genes in the gene set for which eQTLs are available. P—Nominal P value of the hypergeometric test for enrichment. FDR—Bonferroni-corrected P value for testing seven hallmark pathways, and separately, 383 biological process pathways, meeting the requirement of containing at least 3 genes of interest. Locus—Name of the locus which contains the gene expression signals. Genes—Genes which colocalize with the GWAS signal at the locus and are present in the gene set. Cluster—Groups 1 to 8 based on kmeans clustering of all genes within each gene set.

Supplementary Data 8

Description: Multivariate MR leave-one-out sensitivity analysis. Exposure—The GWAS statistics used as exposure in the MR analysis. Excluding SNP— Inverse variance weighted MR analysis without the specified SNP. SNP Label—Nearest gene to the SNP. Beta—Coefficient of the effect of the exposure on the outcome (our multivariate ageing GWAS). SE—Standard Error. P—Nominal two-sided P value of the effect estimate.

Supplementary Data 9

Description: Classification of associations from GWAS catalog and PhenoScanner into broad categories. Category—Disease category; Trait—Name of the trait associated with the locus; PMID—PubMed identification number, where UKBB_Neale refers to the analysis of UK Biobank traits by Neale Lab (Abbott et al., 2018). Loci—Names of the loci which contain a lead SNP or close proxy (r2 EUR > 0.6) which show a genome-wide significant association with the trait.
