# Supplementary material for: Multivariate genomic scan implicates novel loci and haem metabolism in human ageing
Source: Nat Commun. 2020 Jul 16;11:3570. doi: 10.1038/s41467-020-17312-3 (PMC7366647; doi:10.1038/s41467-020-17312-3)
Supplement: Supplementary file 13 — Reporting Summary [file 41467_2020_17312_MOESM13_ESM.pdf]

nature research

Corresponding author(s): Paul Timmers, Peter Joshi, Joris Deelen

Last updated by author(s): May 26, 2020

Reporting Summary

Nature Research wishes to improve the reproducibility of the work that we publish. This form provides structure for consistency and transparency in reporting. For further information on Nature Research policies, see Authors & Referees and the Editorial Policy Checklist .

Statistics

For all statistical analyses, confirm that the following items are present in the figure legend, table legend, main text, or Methods section.

n/a	Confirmed	
☐	☒	The exact sample size (*n*) for each experimental group/condition, given as a discrete number and unit of measurement
☐	☒	A statement on whether measurements were taken from distinct samples or whether the same sample was measured repeatedly
☐	☒	The statistical test(s) used AND whether they are one- or two-sided<br>*Only common tests should be described solely by name; describe more complex techniques in the Methods section.*
☐	☒	A description of all covariates tested
☐	☒	A description of any assumptions or corrections, such as tests of normality and adjustment for multiple comparisons
☐	☒	A full description of the statistical parameters including central tendency (e.g. means) or other basic estimates (e.g. regression coefficient) AND variation (e.g. standard deviation) or associated estimates of uncertainty (e.g. confidence intervals)
☐	☒	For null hypothesis testing, the test statistic (e.g. *F*, *t*, *r*) with confidence intervals, effect sizes, degrees of freedom and *P* value noted<br>*Give P values as exact values whenever suitable.*
☒	☐	For Bayesian analysis, information on the choice of priors and Markov chain Monte Carlo settings
☒	☐	For hierarchical and complex designs, identification of the appropriate level for tests and full reporting of outcomes
☐	☒	Estimates of effect sizes (e.g. Cohen's *d*, Pearson's *r*), indicating how they were calculated

Our web collection on statistics for biologists contains articles on many of the points above.

Software and code

Policy information about availability of computer code

Data collection	No software was used to collect the data.
Data analysis	R v3.6.0 (General statistics); RegScan v0.5 (GWAS regression); R package "survival" v3.1-7 (Cox modelling); LD-score regression v1.0.0 (Genetic correlations); R package "MultiABEL" v1.1-6 (MANOVA); R package "meta" v4.9-7 (Meta-analysis sensitivity); SMR-HEIDI v1.02 (Colocalisation); R package "TwoSampleMR" v0.5.1 (Univariate MR); R package "MendelianRandomization" v0.4.2 (Multivariate MR)

For manuscripts utilizing custom algorithms or software that are central to the research but not yet described in published literature, software must be made available to editors/reviewers. We strongly encourage code deposition in a community repository (e.g. GitHub). See the Nature Research guidelines for submitting code & software for further information.

Data

Policy information about availability of data

All manuscripts must include a data availability statement. This statement should provide the following information, where applicable:

- Accession codes, unique identifiers, or web links for publicly available datasets
- A list of figures that have associated raw data
- A description of any restrictions on data availability

The healthspan, parental lifespan, and longevity GWAS summary statistics are available from OpenAIRE (DOI: 10.5281/zenodo.1302861), Edinburgh DataShare (DOI: 10.7488/ ds/2463), and the longevity genomics website (https://www.longevitygenomics.org/downloads), respectively. The multivariate GWAS summary statistics generated in this study are available from Edinburgh DataShare with the identifier https://doi.org/10.7488/ds/2793. The various summary statistics used to calculate genetic correlations are available from GeneAtlas (http://geneatlas.roslin.ed.ac.uk/), NealeLab (http://www.nealelab.is/uk-biobank), or their respective publications. The lists of SNP-trait associations are available from the GWAS catalog (https://www.ebi.ac.uk/gwas/) and PhenoScanner (http:// www.phenoscanner.medschl.cam.ac.uk/). The hallmark and biological process gene sets are available from the Molecular Signatures Database (https://www.gsea-msigdb.org/). Source data for figures in this study are available in the supplementary documents and upon request from the corresponding author.

Field-specific reporting

Please select the one below that is the best fit for your research. If you are not sure, read the appropriate sections before making your selection.

- [x] Life sciences
- [ ] Behavioural & social sciences
- [ ] Ecological, evolutionary & environmental sciences

For a reference copy of the document with all sections, see nature.com/documents/nr-reporting-summary-flat.pdf

Life sciences study design

All studies must disclose on these points even when the disclosure is negative.

Sample size	Sample size was fixed, determined by the size of the published datasets (Healthspan = 300,477) [Zenin 2019] (Parental lifespan = 1,012,240) [Timmers 2019] (Longevity cases = 11,262; controls = 25,483) [Deelen 2019]. The size of the age-stratified survival analysis in UK Biobank was determined by the number of genomically British individuals with genotypic information in this cohort (N = 409,728) [Bycroft 2018].
Data exclusions	In the UK Biobank analysis, we excluded adopted subjects, subjects with two parents who died before age 40, subjects who did not provide information on parental age, subjects who had withdrawn consent, and one of each set of related subjects. This is necessary to correctly perform a kin-cohort analysis on parental survival [Timmers 2019]
Replication	At the time of writing, the datasets we used contain almost all European-ancestry individuals and nonagarians with genetic information and healthspan/lifespan/longevity measurements. While replication in other cohorts is desirable, we are unaware of any samples large enough to reproduce our findings. However, we attempted to provide some evidence of reproducibility by looking up loci of interest in the GWAS catalog for proxy measures of ageing, such as cardiovascular traits, and found most loci do indeed associate with one or more diseases. We also found many genes linked to the newly discovered LINC02513 locus were previously found to change their expression with age, providing further biological plausibility. Despite this supporting evidence, we acknowledge need for formal replication of newly discovered loci in the manuscript text.
Randomization	No group allocation was performed in this study.
Blinding	No group allocation was performed in this study.

Reporting for specific materials, systems and methods

We require information from authors about some types of materials, experimental systems and methods used in many studies. Here, indicate whether each material, system or method listed is relevant to your study. If you are not sure if a list item applies to your research, read the appropriate section before selecting a response.

Materials & experimental systems

n/a	Involved in the study
[x]	[ ] Antibodies
[x]	[ ] Eukaryotic cell lines
[x]	[ ] Palaeontology
[x]	[ ] Animals and other organisms
[ ]	[x] Human research participants
[x]	[ ] Clinical data

Methods

n/a	Involved in the study
[x]	[ ] ChIP-seq
[x]	[ ] Flow cytometry
[x]	[ ] MRI-based neuroimaging

Human research participants

Policy information about studies involving human research participants

Population characteristics	Characteristics of the healthspan, parental lifespan, and longevity study populations are described in their original publications, see [Zenin 2019], [Timmers 2019], and [Deelen 2019]. Sample descriptives for our age-stratified UK Biobank population can be found in Supplementary Table 4.
Recruitment	Recruitment of participants is described in the original studies. See [Zenin 2019], [Timmers 2019], and [Deelen 2019]. Recruitment of UK Biobank individuals is described in [Bycroft 2018].
Ethics oversight	The study was approved by UK Biobank under application 19655.

Note that full information on the approval of the study protocol must also be provided in the manuscript.
